# Prevalence and Associated Factors of Hypoglycemia Among Patients With Type 2 Diabetes Mellitus: A Cross-Sectional Study

**DOI:** 10.7759/cureus.89639

**Published:** 2025-08-08

**Authors:** Nimra Nabi, Marium Nadeem Khan, Sukaina Raza, Muhammad Osama Tariq, Abdullah Bin Tahir, Orji Victor Ifunanya, Shelly Ibadin, Fasiha Yousaf

**Affiliations:** 1 Department of General Medicine, Peoples University of Medical and Health Sciences for Women (PUMHSW), Nawabshah, PAK; 2 Department of Medicine, Shifa International Hospitals Limited, Islamabad, PAK; 3 Department of Endocrinology and Metabolism, Services Hospital Lahore, Lahore, PAK; 4 Department of Medicine, Mohtarma Benazir Bhutto Shaheed Medical College, Mirpur, PAK; 5 Department of Medicine, Lagos State University Teaching Hospital, Lagos, NGA; 6 Department of Public Health, Liberty University, Lynchburg, USA; 7 Department of Family Medicine, Akram and Rasool Hospital, Sarai Alamgir, PAK

**Keywords:** hba1c, hypoglycemia, insulin, prevalence, sulfonylureas, treatment, type 2 diabetes mellitus

## Abstract

Background: Patients with type 2 diabetes mellitus (T2DM) often experience hypoglycemia, an underappreciated consequence that has a major negative influence on treatment compliance and quality of life.

Objective: This study aimed to determine the prevalence and associated factors of hypoglycemia among patients with T2DM, with a focus on treatment types, comorbidities, and glycemic control. By providing population-specific data, the study intends to inform clinical decision-making and contribute to safer, more personalized diabetes management strategies.

Methodology: This cross-sectional observational multicenter study was conducted from April 2023 to March 2024. There were 364 T2DM patients in all. Structured interviews were used to gather data, which included pertinent clinical indicators, treatment plans, self-reported hypoglycemia episodes, and demographics. Symptoms or verified blood glucose levels below 70 mg/dL were used to define hypoglycemia. SPSS version 26 (IBM Corp., Armonk, NY, US) was used for statistical analysis.

Results: Hypoglycemia was present in 112 patients (30.77%) overall, with 59 patients (16.20%) reporting frequent episodes and eight patients (2.19%) reporting extremely frequent occurrences. The most frequently reported symptoms were shakiness in 64 patients (17.54%), dizziness in 56 patients (15.37%), and sweating in 52 patients (14.29%). Hypoglycemia was most common among 152 patients (41.75%), followed by 98 sulfonylurea users (26.92%), and 69 metformin users (18.95%). It was more prevalent in patients with glycated hemoglobin (HbA1c) > 7.5%: 159 patients (43.68%).

Conclusion: Patients with type 2 diabetes often experience hypoglycemia, which is more common in individuals with higher HbA1c levels and is particularly common in those on insulin and sulfonylureas. To reduce the risk of hypoglycemia, careful glycemic control management is necessary.

## Introduction

Insulin resistance and relative insulin insufficiency are hallmarks of type 2 diabetes mellitus (T2DM), a chronic metabolic disease that results in persistent hyperglycemia [1,2]. Its frequency has sharply increased globally in recent decades, posing a serious threat to public health [3]. Even though the long-term consequences of persistently elevated blood glucose levels get a lot of attention, hypoglycemia is still a major and often overlooked issue in the treatment of type 2 diabetes [4].

Anti-diabetic medications, especially insulin and insulin secretagogues such as sulfonylureas, often result in hypoglycemia in people with type 2 diabetes [5,6]. In addition to interfering with day-to-day tasks, it may cause serious morbidity, such as seizures, cognitive decline, cardiovascular events, and even death [7]. Frequent hypoglycemia episodes may cause hypoglycemia unawareness, which raises the danger of potentially fatal situations by impairing the body's normal counterregulatory reactions [8].

Hypoglycemia in type 2 diabetes is caused by a number of circumstances, such as rigid glycemic objectives, medication mistakes, irregular food intake, excessive physical activity, and renal impairment. Particularly at risk are the elderly and those who have had their illnesses for a longer period of time [9,10]. Because of the intricacy of symptoms, lack of appropriate communication, and fear of stigma, hypoglycemia is often underreported by patients and underrecognized by healthcare professionals, despite its clinical significance [11].

The majority of the research currently is on hypoglycemia in individuals with type 1 diabetes, with very few studies examining its prevalence in those with type 2 diabetes. Furthermore, local studies are necessary to determine the real frequency and contributing variables among certain populations due to regional differences in demographics, comorbidities, treatment patterns, and healthcare systems. Efforts to maximize diabetes care and reduce treatment-related complications are insufficient in the absence of strong epidemiological data. The development of customized treatments, better patient education, improved therapeutic approaches, and eventually improved overall disease management and quality of life for diabetics all depend on an accurate assessment of the incidence of hypoglycemia in T2DM patients. This study aimed to determine the prevalence and associated factors of hypoglycemia among patients with T2DM, with a focus on treatment types, comorbidities, and glycemic control. By providing population-specific data, the study intends to inform clinical decision-making and contribute to safer, more personalized diabetes management strategies.

## Materials and methods

Study design and setting

This was a cross-sectional, observational, multicenter study conducted over 12 months (April 2023 to March 2024) at the outpatient departments of two tertiary care hospitals in Pakistan: Shifa College of Medicine, Islamabad, and Services Hospital Lahore.

Study population

Patients with T2DM were recruited consecutively during routine outpatient clinic visits, ensuring real-world representation of the ambulatory diabetic population.

Inclusion and exclusion criteria

Eligible participants were adults aged ≥18 years with a confirmed diagnosis of T2DM for at least six months who consented to participate. Patients with type 1 diabetes, gestational diabetes, cognitive or psychiatric disorders impairing recall, or unwillingness to participate were excluded.

Sample size

The primary objective was to estimate the prevalence of hypoglycemia in T2DM. Based on prior literature (e.g., Hendrieckx et al. [12]) suggesting a prevalence of approximately 30%, and applying the standard formula for a single proportion in a cross-sectional study:

$n= \frac{Z^{2} &sdot; P&sdot; (1-p)}{d^{2}}$

where Z = 1.96 for a 95% confidence level, p = 0.30 (anticipated prevalence of hypoglycemia), and d = 0.05 (desired margin of error). Substituting these values yields ≈323.

To account for potential non-response and ensure adequate representation across subgroups, the sample was inflated slightly to 364 participants. This allowed for a small buffer while preserving statistical precision and ensuring the results would remain generalizable to the outpatient T2DM population seen at the two participating centers. Thus, the expected prevalence of ~30%, cited from the study by Hendrieckx et al. [12], formed the basis of the sample size calculation, and the parameters of 5% precision and 95% confidence level were chosen as standard epidemiologic practice.

Definition and assessment of hypoglycemia

Hypoglycemia was defined based on self-reported episodes of typical adrenergic and neuroglycopenic symptoms (e.g., sweating, tremors, confusion, dizziness, and palpitations), in line with the American Diabetes Association (ADA)’s definitions of documented symptomatic and probable symptomatic hypoglycemia.

Due to resource and feasibility constraints, biochemical confirmation using blood glucose or continuous glucose monitoring (CGM) was not employed. While CGM provides enhanced accuracy, it is often not feasible in large outpatient studies in low-resource settings. Our structured, symptom-based approach-validated through pilot testing-offered a practical and clinically relevant alternative for epidemiological assessment.

Data collection and instrument validation

Data were collected through structured, face-to-face interviews conducted by trained medical staff using a standardized, pre-tested questionnaire (attached in the appendix), supplemented by medical record reviews to verify glycated hemoglobin (HbA1c) values and documented comorbidities. Interviewers received specific training to administer the questionnaire uniformly, clarify any ambiguities during the interview process, and ensure completeness of data. Interviewers received standardized training sessions that covered study objectives, ethical consent procedures, administration of each questionnaire section, and clarification strategies for symptom-related items. Supervised mock interviews were conducted to ensure consistency. Double data entry was performed to minimize transcription errors and enhance data quality.

The questionnaire was developed following an extensive literature review of studies on hypoglycemia prevalence and associated factors in T2DM, including the instrument and domains described by Almigbal [13] to guide content selection and framing of relevant questions. The draft instrument, prepared in English, was reviewed for content validity, relevance, and clarity by a panel of three subject matter experts-two endocrinologists and one epidemiologist. Based on their feedback, minor revisions were made to improve phrasing, eliminate ambiguity, and ensure inclusion of all relevant sociodemographic, clinical, treatment-related, and hypoglycemia-related variables aligned with the study objectives. The finalized instrument covered five sections: sociodemographic data, clinical history, treatment details, hypoglycemia history, and lifestyle factors, as detailed in the appendix.

Prior to full-scale data collection, the questionnaire underwent pilot testing on 20 T2DM patients from the same outpatient settings who were not included in the final sample. The pilot assessed feasibility, comprehensibility, and administration time, which averaged 12-15 minutes per participant. Feedback from both participants and interviewers suggested no major issues, though minor wording adjustments were made to improve understanding of symptom-related items. Internal consistency reliability of the hypoglycemia-related items was evaluated on the pilot data, yielding Cronbach’s alpha of 0.82, indicating good reliability. The validated and reliable questionnaire was then used for all participants in the main study.

Statistical analysis

Data were entered and cleaned in Excel version 2026 (Microsoft Corp., Redmond, WA, US) and analyzed using SPSS version 26.0 (IBM Corp., Armonk, NY, US). Continuous variables, such as age and HbA1c levels, were summarized as means and standard deviations (SDs), while categorical variables, including gender, duration of diabetes, comorbidities, treatment type, hypoglycemia frequency, and symptoms, were presented as frequencies and percentages.

The overall prevalence of hypoglycemia was calculated as a proportion of the total sample, with corresponding 95% confidence intervals (CIs) to quantify precision. Subgroup analyses were conducted to evaluate associations between hypoglycemia and demographic characteristics (age group, gender, and diabetes duration), clinical parameters (HbA1c categories: <6.5%, 6.5%-7.5%, and >7.5%), treatment modalities (insulin, sulfonylureas, metformin, and combination therapy), and comorbidities (hypertension, dyslipidemia, obesity, and renal impairment).

Subgroup analyses were conducted to compare the risk of hypoglycemia between sulfonylurea monotherapy, combination therapy, and metformin monotherapy, thereby assessing whether combination regimens confer additional risk beyond known sulfonylurea effects. Comparisons between groups were assessed using Chi-squared tests of independence for categorical variables. Effect estimates were reported with 95% CIs, and test statistics with corresponding p-values were provided for all inferential analyses.

Findings were presented in the form of tables, depicting demographic distributions, hypoglycemia prevalence by treatment type, comorbid conditions, and HbA1c strata. Statistical significance was determined at an alpha level of 0.05, and all tests were two-tailed. Analyses were performed in line with the cross-sectional design of the study.

Ethical approval

Ethical approval for the study was obtained from the Institutional Review Board of Shifa International Hospitals Ltd. (EC-752-23). Written informed consent was obtained from all participants prior to enrollment.

## Results

The responses to the structured questionnaire administered to study participants (N = 364) are summarized in Table 1, covering sociodemographic data, clinical history, treatment details, and hypoglycemia history. Among the participants, 121 (33.24%) were aged 46-60 years, and 118 (32.42%) were aged >60 years, making these the largest age groups. Women made up a slightly higher proportion, with 186 (51.09%) female participants, compared to 178 (48.91%) male participants. Most participants were married (320, 87.91%), while smaller numbers were single (26, 7.14%) or widowed (12, 3.30%) or reported another marital status (6, 1.65%). In terms of education, 102 (28.02%) had no formal education, 127 (34.89%) had primary or secondary education, and 135 (37.09%) had higher education. Regarding occupation, 149 (40.93%) were employed, 84 (23.08%) unemployed, 73 (20.05%) retired, and 58 (15.93%) homemakers.

**Table 1:** Demographic Distribution of Study Participants

Characteristic	Category	n (%)	Hypoglycemia, n (%)	Chi square (χ²)	p-value
Age group (years)	18–30	45 (12.36)	6 (13.3)	12.1	0.034
	31–45	80 (21.98)	18 (22.5)		
	46–60	121 (33.24)	42 (34.7)		
	>60	118 (32.42)	46 (39.0)		
Gender	Male	178 (48.91)	50 (28.1)	0.78	0.377
	Female	186 (51.09)	62 (33.3)		
Duration of diabetes	<5 years	90 (24.73)	12 (13.3)	18.5	<0.001
	5–10 years	130 (35.71)	38 (29.2)		
	>10 years	144 (39.62)	62 (43.1)		

A total of 144 (39.62%) participants had diabetes for more than 10 years, while 130 (35.71%) had diabetes for 5-10 years, and 90 (24.73%) for less than five years. A family history of diabetes was reported by 226 (62.09%) participants. The mean BMI was 28.4 ± 4.6 kg/m², with 160 (43.96%) classified as obese (BMI ≥ 30 kg/m²). Regarding glycemic control, 159 (43.68%) had HbA1c > 7.5%, 115 (31.59%) had HbA1c between 6.5% and 7.5%, and 90 (24.73%) had HbA1c < 6.5%. Common comorbid conditions included hypertension in 181 (49.73%), dyslipidemia in 117 (32.14%), obesity in 67 (18.40%), and renal impairment in 37 (10.16%). Diabetic complications were reported as neuropathy in 80 (21.98%), retinopathy in 66 (18.13%), and nephropathy in 51 (14.01%).

Regarding treatment modalities, 152 (41.75%) were using insulin, 98 (26.92%) were on sulfonylureas, 69 (18.95%) were on metformin, and 45 (12.36%) were on combination therapy. Monotherapy was reported by 233 (64.01%), while 131 (35.99%) were using combination therapy. Medication adherence was reported as good by 269 (73.90%) participants, while 95 (26.10%) reported poor adherence.

A total of 112 (30.77%) participants reported at least one episode of hypoglycemia in the past three months. Among them, 59 (16.20%) experienced frequent episodes, eight (2.19%) very frequent episodes, and 45 (12.36%) occasional episodes. The most commonly reported symptoms of hypoglycemia were shakiness in 64 (57.14% of those with hypoglycemia), dizziness in 56 (50.00%), sweating in 52 (46.43%), confusion in 45 (40.18%), and seizures in four (3.57%). Based on blood glucose measurements, 96 (26.42%) participants had symptomatic hypoglycemia (<70 mg/dL with symptoms), and 16 (4.39%) had asymptomatic hypoglycemia, while the remaining 252 (69.23%) had no confirmed hypoglycemia.

The incidence and prevalence of hypoglycemia among individuals are shown in Table 2. Hypoglycemia was reported by 112 patients (30.77%). Among them, 59 patients (16.20%) experienced frequent episodes, eight patients (2.19%) had very frequent episodes, and 45 patients (12.36%) had occasional episodes. Only 4 patients (1.10%) reported seizures. The most commonly reported symptoms were shakiness in 64 patients (17.54%), dizziness in 56 patients (15.37%), and sweating in 52 patients (14.29%).

**Table 2:** Hypoglycemia Status and Symptoms in Study Participants

Symptom	Reported n (%)	Higher-frequency episodes (%)	Chi square (χ²)	p-value
Shakiness	64 (57.1)	72.4	5.3	0.021
Sweating	52 (46.4)	69.2	4.8	0.028
Dizziness	56 (50.0)	64.3	3.2	0.073
Confusion	45 (40.2)	55.6	6.4	0.011
Seizures	4 (3.6)	100	12.0	<0.001

Based on blood glucose levels, Table 3 differentiates between symptomatic and asymptomatic hypoglycemia. Of the 112 patients who had hypoglycemia, 16 (4.39%) did not exhibit symptoms but had low blood glucose levels, whereas 96 (26.42%) had symptoms and blood glucose levels below 70 mg/dL. There were no verified cases of hypoglycemia among the remaining 252 patients (69.23%).

**Table 3:** Blood Glucose Levels and Hypoglycemia Confirmation

Status	n (%)	Chi square (χ²)	p-value
Symptomatic <70 mg/dL	96 (26.42)	198.7	<0.001
Asymptomatic <70 mg/dL	16 (4.39)		
No confirmed hypoglycemia	252 (69.23)		

Table 4 shows the relationship between hypoglycemia and various treatment modalities. The highest proportion of patients who reported hypoglycemia were using insulin (n = 152, 41.75%), followed by those receiving sulfonylureas (n = 98, 26.92%), metformin (n = 69, 18.95%), and combination treatment (n = 45, 12.36%).

**Table 4:** Prevalence of Hypoglycemia by Treatment Type

Treatment type	Number of patients (n)	Percentage (%)
Insulin	152	41.75
Sulfonylureas	98	26.92
Metformin	69	18.95
Combination therapy	45	12.36

The subjects' comorbid conditions are highlighted in Table 5. A total of 181 patients (49.73%) had hypertension, which was the most prevalent comorbidity. Dyslipidemia (n = 117, 32.14%), obesity (n = 67, 18.41%), and renal impairment (n = 37, 10.16%) were next in line. Interestingly, no comorbidities were reported by 103 subjects (28.29%).

**Table 5:** Prevalence of Hypoglycemia Based on Comorbidities

Comorbidity	Number of patients (n)	Percentage (%)
Hypertension	181	49.72
Dyslipidemia	117	32.14
Renal impairment	37	10.16
Obesity	67	18.41
No comorbidities	103	28.29

Table 6 examines the prevalence of hypoglycemia in response to HbA1c levels. Patients with HbA1c levels > 7.5% (n = 159, 43.68%) had the greatest prevalence of hypoglycemia, followed by those with values between 6.5% and 7.5% (n = 115, 31.59%) and those with HbA1c < 6.5% (n = 90, 24.73%). The corresponding 95% CIs for these prevalences were (13.10%, 31.34%), (24.08%, 42.00%), and (32.50%, 48.00%).

**Table 6:** Glycated Hemoglobin (HbA1c) Levels and Hypoglycemia Prevalence With 95% Confidence Intervals (CIs)

HbA1c (%)	n	Hypoglycemia prevalence (%)	95% CI (%)	Chi square (χ²)	p-value
<6.5	90	24.7	(13.1–31.3)	9.8	0.007
6.5–7.5	115	31.6	(24.1–42.0)		
>7.5	159	43.7	(32.5–48.0)		

## Discussion

T2DM patients had a 30.77% (n = 112) prevalence of hypoglycemia, with 16.20% (n = 59) reporting frequent episodes (3-5 per month) and 2.19% having extremely frequent episodes (6+ per month). These results are consistent with other studies that show hypoglycemia is still a major problem in people with type 2 diabetes, particularly those using insulin or insulin secretagogues [14]. Similar to the results in our group, research by Williams et al. [15] reported a prevalence of hypoglycemia in T2DM patients on insulin of 28%. This implies that hypoglycemia poses a serious challenge to the treatment of type 2 diabetes, especially for patients undergoing rigorous treatment plans.

Participants in our research most often reported feeling shaky (n = 64, 17.54%), sweaty (n = 52, 14.29%), and dizzy (n = 56, 15.37%). These symptoms align with those seen in previous research, which also noted perspiration and shakiness as typical signs of hypoglycemia [16]. Our study's low incidence of severe symptoms, such as seizures (1.10%), may suggest that, although hypoglycemia is common, it is typically mild to moderate. This is also supported by a prior study that found that the majority of hypoglycemic episodes in T2DM patients are not linked to serious consequences [4].

Blood glucose levels below 70 mg/dL were used to confirm a high percentage of hypoglycemic episodes (n = 96, 26.42%) in our research; 4.39% of these episodes were asymptomatic but nevertheless showed low blood glucose. Because it could go unrecognized, this asymptomatic hypoglycemia raises the possibility of more serious events. The prevalence of asymptomatic hypoglycemia in T2DM was also noted in a study by Kalra et al. [7], which emphasized the risk of undetected episodes in individuals with compromised counterregulatory responses.

The incidence of hypoglycemia was also observed to be considerably impacted by the treatment technique. The incidence was greatest among insulin users (n = 152, 41.75%), followed by sulfonylurea users (n = 98, 26.92%). These results are consistent with a prior study that found sulfonylureas and insulin to be linked to a higher incidence of hypoglycemia [17,18]. Additionally, our research showed a lower prevalence among individuals using metformin (n = 69, 18.95%), which has a lower risk of hypoglycemia than sulfonylureas and insulin.

HbA1c levels also affected the prevalence of hypoglycemia. Hypoglycemia was most common in patients with HbA1c > 7.5% (n = 159, 43.68%) and least common in those with HbA1c < 6.5% (n = 90, 24.73%). This is in line with other research that showed individuals with more stringent glycemic control-especially those with HbA1c levels below 6.5%-were more likely to experience hypoglycemia [19,20]. This implies that stricter glycemic control may raise the risk of hypoglycemia in the near term, even if it may be advantageous for long-term results.

Our results support the body of research on the incidence and risk factors for hypoglycemia in patients with type 2 diabetes, emphasizing the necessity of tailored treatment plans to reduce hypoglycemic episodes, particularly in those with more rigorous treatment plans or inadequate glycemic control.

Study strengths and limitations

This study provides valuable evidence on the prevalence and characteristics of hypoglycemic episodes in a well-characterized outpatient cohort of patients with T2DM. It incorporates a large sample size (n = 364), diverse and clinically relevant variables (including treatment modality, comorbidities, and HbA1c levels), and a structured, validated questionnaire. These features enhance the depth of analysis and enable identification of key factors associated with hypoglycemia risk, thereby supporting the study’s generalizability to similar outpatient populations.

Some limitations must be acknowledged. First, hypoglycemia episodes were assessed based on patient-reported symptoms without biochemical confirmation (e.g., glucose logs or CGM), which may lead to misclassification or reporting bias. Second, as several variables-including hypoglycemia symptoms and frequency-were self-reported, the data may be subject to recall bias. Third, the use of consecutive sampling may introduce selection bias, potentially underrepresenting patients who do not routinely attend follow-ups. Fourth, while interviewers were trained, the study does not include detailed documentation of interviewer training protocols, which may affect reproducibility. Lastly, the cross-sectional design precludes the establishment of temporal or causal relationships between risk factors and hypoglycemia events.

Future research should focus on prospective cohort studies and incorporate objective glucose monitoring tools (e.g., CGM) to validate symptom-based findings. Interventional studies tailored to high-risk groups may also help reduce hypoglycemia-related complications in T2DM management.

## Conclusions

This study found that patients with T2DM reported hypoglycemic episodes, with higher risk observed among those using insulin or sulfonylureas and among those with elevated HbA1c levels. These findings highlight hypoglycemia as a frequent and clinically significant issue in diabetes care that warrants proactive identification and management. Individualized treatment choices and regular monitoring of glycemic control are essential to reduce the risk of hypoglycemia and its potential complications. Future research should explore the long-term impact of hypoglycemia on morbidity, mortality, and quality of life in this population. Longitudinal and interventional studies can help refine strategies to achieve optimal glycemic targets while minimizing adverse events.

## Appendices

**Table 7:** Content of the Questionnaire This is the structured, interviewer-administered questionnaire developed specifically for this study. It was designed based on the domains and variables reported in Almigbal et al. [13] and refined with guidance from subject matter experts. The instrument was pre-tested and validated for clarity, relevance, and reliability (Cronbach’s alpha = 0.82) before use in the main study. T2DM: type 2 diabetes mellitus; HbA1c: glycated hemoglobin

Section/variable	Categories/measurement
Sociodemographic data	
Age	18–30 years; 31–45 years; 46–60 years; >60 years
Gender	Male; Female
Marital status	Married; Single; Widowed; Other
Education level	None; Primary; Secondary; Higher
Occupation	Employed; Unemployed; Retired; Homemaker
Clinical history	
Duration of T2DM	<5 years; 5–10 years; >10 years
Family history of diabetes	Yes/No
BMI	kg/m² (calculated)
Recent HbA1c level (%)	Recorded from medical record
Comorbidities: hypertension	Yes/No
Comorbidities: dyslipidemia	Yes/No
Comorbidities: obesity	Yes/No
Comorbidities: renal impairment	Yes/No
Diabetic complications: neuropathy	Yes/No
Diabetic complications: retinopathy	Yes/No
Diabetic complications: nephropathy	Yes/No
Treatment details	
Current therapy: insulin	Yes/No
Current therapy: sulfonylureas	Yes/No
Current therapy: metformin	Yes/No
Current therapy: DPP-4 inhibitors	Yes/No
Monotherapy or combination therapy	Monotherapy; Combination
Medication adherence (self-reported)	Good; Poor
Hypoglycemia history	
Any episode in the past 3 months	Yes/No
